# Thoracic Epidural Analgesia for Severe Acute Pancreatitis: Quo Vadis Intensivist?

**DOI:** 10.5005/jp-journals-10071-23117

**Published:** 2019-02

**Authors:** Deepak Govil, Mozammil Shafi

**Affiliations:** 1,2 Institute of Anesthesia and Critical Care, Medanta The Medicity, Gurugram, Haryana, India

## Abstract

**How to cite this article:** Govil D, Shafi M. Thoracic Epidural Analgesia for Severe Acute Pancreatitis: Quo Vadis Intensivist? Indian J of Crit Care Med 2019;23(2):59-60.

**A**cute pancreatitis (AP) is a common gastrointestinal disorder requiring intensive care unit admission. Severe form of this disease is associated with substantial mortality and morbidity and often require prolonged hospitalisation^1^. Although pathogenesis of AP is not clearly understood, several mechanisms have been postulated. Intra-acinar activation of pancreatic enzymes and resulting local and systemic inflammation plays an important part in disease process. Microcirculatory injury due to damage of vascular endothelium and interstitium from activated pancreatic enzymes is an early step in disease pathogenesis^2^. Severe epigastric abdominal pain is earliest and most common symptom of AP and adequate pain management is an integral component of disease management^3^. Intravenous opioid analgesics are safe and commonly used method of pain control.

Epidural analgesia (EA) is one of the most effective method of pain control during perioperative period. Apart from excellent pain control, epidural analgesia is also associated with reduction in post-operative pulmonary, cardiovascular and thrombotic complications, helps in early mobilisation, and decreases incidence of ileus, after major abdominal surgeries^4–5^. Although role of EA in management of AP is not very well defined many centres around the world have been using it for acute pain control^6–7^. EA has been found useful in improving pancreatic perfusion, decreasing severity of disease and mortality in number of animal studies^8–10^. Improvement in pancreatic microcirculation is the most consistent finding possibly due to sympathetic blockade and resulting vasodilatation induced by EA. Even though randomized placebo control trials of EA in AP are lacking, few observational studies are available. Bernhardt *etal* and Jabaudon *etal*. in their respective observational studies have clearly documented safety of EA in management of AP^6,7^. In a randomized pilot study by Sadowski *etal*. comparing EA with systemic analgesia, improvement in pancreatic microcirculation as evaluated by CT scan (On admission and 72 hours later) was observed in 43% in EA group and 7% in Control group^11^.

In the present issue of this journal Dr Tyagi *et al.*^12^ conducted a randomized pilot study evaluating outcome of Thoracic epidural analgesia in patients of severe pancreatitis. Patients were divided between two groups and they either received Thoracic EA (TE, n = 16) or did not receive Thoracic EA (NTE, n = 16). Severe pancreatitis was diagnosed by surgeons using International Association of Pancreatology (IAP)/American Pancreatic Association (APA) criteria. Primary end point was aggregate Sequential Organ Failure Assessment (SOFA) score from admission day to day 3. Aggregate SOFA score was calculated by adding worst score of each organ system during ICU stay. Authors also documented few secondary end points like in hospital mortality, length of intensive care stay and duration of mechanical ventilation. Values of Inflammatory mediators like serum procalcitonin, C-reactive protein, tumor necrosis factor-*a* and interleukin-6 (IL-6) were also obtained for both groups. There was no statistically significant difference seen in primary outcome between two groups (aggregate SOFA score TE and group NTE (5 [2 – 6] vs. 3 [2 – 4]; *P* = 0.379). Mean difference in aggregate SOFA score was 0.4 with standard error (95% CI) of −1.7 (−3 to 3.9). In secondary end points, mortality was lower in TE group but did not reach statistical significance. Other secondary outcomes were not statistically different between two groups. All Inflammatory markers decreased in TE group but values of only procalcitonin reached statistical significance (p=0.010). An important point worth mentioning is that despite randomization, SOFA score on day 0 was higher for TE group (4 vs 2).

This is one of the few randomized trials evaluating meaningful clinical outcome in patients of severe pancreatitis managed with epidural analgesia. Result of the present trial is in contrast to many experimental studies discussed above. Although authors noted an improved daily SOFA score in TE group, aggregate score did not reach statistical significance. One possible explanation is, baseline discrepancy in SOFA score between two groups despite randomization. Another possible reason may be the study is not powered enough to detect any meaningful clinical difference if it exists. Level of inflammatory biomarkers decreased in epidural group, but only procalcitonin reached statistical significance. This point is worth considering given the role of inflammation in perpetuating organ dysfunction in pancreatitis. The present study reemphasised the finding from previous trials, pertaining to safety of epidural analgesia by experienced clinicians and staffs. In a nutshell, this is a meticulous and well conducted study, but needs validation in a larger well designed randomized trial. The epidural analgesia for pancreatitis (EPIPAN) trial is a multicenter randomized control trial, comparing epidural analgesia with usual care in patients of acute pancreatitis^13^. Primary end point is number of mechanical ventilation free days and many clinically relevant secondary end points including biomarkers are being looked into. This study will give an important insight on role of epidural analgesia in management of AP.
